# Optimizing the Learner’s Role in Feedback: Development of a Feedback-Preparedness Online Application for Medical Students in the Clinical Setting

**DOI:** 10.7759/cureus.38722

**Published:** 2023-05-08

**Authors:** Victoria Blouin, Florence Bénard, Florence Pelletier, Sandy Abdo, Léamarie Meloche-Dumas, Bill Kapralos, Adam Dubrowski, Erica Patocskai

**Affiliations:** 1 Faculty of Medicine, Université de Montréal, Montreal, CAN; 2 General Surgery, Université de Montréal, Montreal, CAN; 3 Health Sciences, Ontario Tech University, Oshawa, CAN; 4 Medical Education and Simulation, maxSIMhealth Group, Ontario Tech University, Oshawa, CAN; 5 Surgical Oncology, Université de Montréal, Montreal, CAN

**Keywords:** online application, clerkship, self-assessment, feedback, teaching, medical education

## Abstract

Feedback is an essential component of medical education, especially during clinical rotations. There is growing interest in learner-related factors that can optimize feedback’s efficiency, including goal orientation, reflection, self-assessment, and emotional response. However, no mobile application or curriculum currently exists to specifically address those factors. This technical report describes the concept, design, and learner-based feedback of an innovative online application, available on mobile phones, developed to bridge this gap.

Eighteen students in their third or fourth year of medical school provided comments on a pilot version of the application. The majority of learners deemed the module relevant, interesting, and helpful to guide reflection and self-assessment, therefore fostering better preparation before an upcoming feedback session. Minor improvements were suggested in terms of content and format.

The learners’ initial positive response supports further efforts to engage in validity and evaluation research. Future steps include modifying the mobile application based on learners’ comments, evaluating its efficacy in a real clinical setting, and clarifying whether it is most beneficial for mid-rotation or end-of-rotation feedback sessions.

## Introduction

Feedback is considered an essential part of medical education and can be defined as “specific information about the comparison between a learner's observed performance and a standard, given with the intent to improve the learner's performance” [1]. The complex process of feedback is affected by many factors related to the task performance itself, the observation of the task, the communication of feedback by the feedback provider, and the interpretation of feedback by the feedback receiver [2]. While observation-related factors and communication-related factors are extensively studied, there is a relative paucity of literature exploring learner-related factors [2].

In recent years, more attention has been given to these learner-related factors influencing feedback efficacy, particularly the theory of goal orientation [3,4]. Learners with a performance-goal orientation seek positive comments to validate their competence, avoid negative judgments [3], and perceive higher risks than benefits from seeking feedback [4]. On the other hand, learners with a learning-goal orientation perceive higher benefits from getting feedback [4], as it constitutes an opportunity to develop their skills and progress towards competency [3]. In turn, the perceived benefits and risks associated with each goal orientation influence the feedback process and affect learners’ feedback-seeking behavior [5]. This learning-goal orientation may be difficult to achieve, considering that performance pressure, including the impression of constant and subjective assessment throughout clinical rotations, is a documented stressor for medical students [6]. However, goal orientation can be influenced. A study by Nussbaum et al. showed that following negative feedback, learners that were led to believe intelligence is malleable tried to improve themselves, while learners who were led to believe intelligence is fixed adopted defensive strategies [7].

Other factors highlighted as having a potential positive effect on feedback received include the learner’s reflection [8] and self-assessment of strengths and weaknesses [9], as well as autonomy [10] and emotional response towards feedback [11]. Literature, therefore, supports training learners in receiving feedback [12,13], although such programs are not widely described in the literature or implemented in current Canadian medical schools’ curriculum.

Feedback plays a key role in learners’ training, especially during clinical rotations, where they receive both punctual formative feedback on a day-to-day basis and summative feedback such as end-of-rotation evaluation. However, feedback in a clinical environment can be limited by barriers including time constraints and suboptimal feedback delivery by supervisors [12]. Informal discussions with medical students also revealed that they do not systematically take the time to appropriately reflect on their goals and performance before summative feedback, which could decrease their active participation in feedback sessions. Empowering learners to better prepare for and handle feedback is therefore essential to allow them to benefit from this learning opportunity [12].

The aim was to develop a practical application, available on learners’ phones or tablets, to foster a better understanding of feedback’s role and help them self-assess in preparation for summative feedback in a clinical setting. To our knowledge, this is the first application destined for medical learners to improve the learner-related factors influencing feedback efficacy. Through a design thinking lens [14], the purpose of this technical report is to describe the development and preliminary user feedback of this innovative mobile application.

## Technical report

Context

The idea of developing an application to enhance learners’ preparation before receiving feedback arose from an informal needs assessment in our institution. Third and fourth-year medical students receiving end-of-the-block summative feedback often did not take the time to reflect before entering the evaluation session, and inaccurate self-assessment was common. This is coherent with current literature on self-assessment, which supports that although this skill is essential in the feedback process, humans perform it poorly [15]. Moreover, summative evaluations are associated with significant anxiety in some learners, generating emotional responses that could decrease their receptivity to feedback. They tended to perceive this evaluation as a simple judgment of their performance, rather than an opportunity to confirm their strengths and identify potential areas of improvement.

During an ideation session, our group suggested a novel approach to improving feedback efficacy: creating an application to address the learner-related factors influencing feedback. The application would be destined for third and fourth-year medical students completing a clinical rotation and would be used to better prepare the learners immediately prior to their end-of-block feedback session. The application is available on computers, tablets, and mobile phones and is meant to be completed in a few minutes, on the day of the evaluation.

The learning application was designed through a collaboration between a clinical team specializing in surgical education, responsible for developing the application’s content based on pertinent literature, and a technical team, responsible for integrating the content onto a digital platform. The clinical team consisted of surgery residents and medical students, under the supervision of the surgical oncologist responsible for the mandatory surgery clinical rotation at the Université de Montréal (Montréal, Canada). The technical team was composed of graduate students and researchers from maxSIMhealth, a research laboratory specialized in simulation-based health professions education from the Ontario Tech University (Oshawa, Canada).

Design

Our application consists of a learning module available on the Gamified Educational Network (GEN), a system developed by the maxSIMhealth team, at Ontario Tech University. GEN is a virtual learning platform designed to enhance self-directed learning. Virtual learning modules can incorporate interactive elements such as progress bars, questions for learners to answer, or discussion boards [16].

The module is divided into distinct sections, each of which must be completed before advancing to the next, with a progress bar indicating the number of remaining sections. Throughout the application, careful consideration was given to wording choices. Active verbs were deliberately used to suggest learners should play an active role in the feedback process rather than simply “receive” the feedback. Words related to evaluation were omitted, when possible, to orient learners towards a learning goal orientation rather than a performance goal orientation. The text was written in French as it is the primary language used in our institution and translated to English for publication purposes.

Content

**Figure 1:**
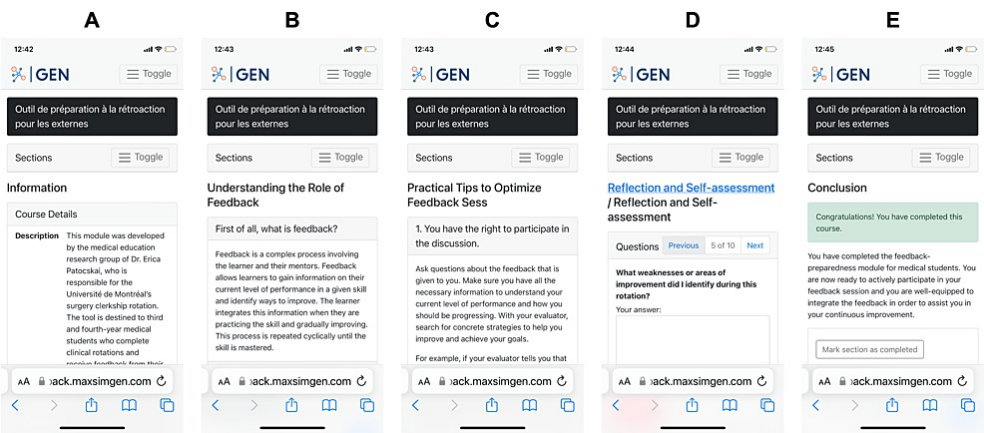
Screenshots of the learning module as shown on a mobile phone. A: Introduction section. B: Understanding the Role of Feedback section. C: Practical Tips section. D: Reflection section. E: Conclusion section.

The module is composed of a total of five sections, as shown in Figure 1. The first one introduces the aim of the learning module and defines its objectives. It also specifies that all data collected in the application is confidential and will not be provided to the faculty. This is followed by three core sections: understanding the role of feedback, tips for the feedback session, and personal reflection questions, all of which are detailed below. The last section is a short conclusion and references. The full content of the learning module is available in Appendix 1 (Table 3).

Core Section 1: Understanding the Role of Feedback

This section is strongly based on learning goal orientation theories [4]. It offers an explanation to learners of how feedback can help them progressively acquire and improve the skills they require to become competent physicians. It therefore encourages them to perceive the feedback session as a learning opportunity, aiming to draw learners towards a learning goal orientation, rather than a performance goal orientation.

Core Section 2: Practical Tips to Optimize Feedback Session

This section offers tips to learners on how to approach their feedback session. Learners are encouraged to verbally engage in discussion with their supervisor during the feedback session and to seek additional comments and clarifications when appropriate. When provided with feedback, learners are advised to identify the main objectives on which they want to focus and specific areas that they would like to improve. Collaborating with their feedback provider in developing strategies to improve those skills is also highly recommended. This aims to empower learners and foster the development of autonomy and self-determination with regard to feedback, as well as supporting goal-setting behaviors, in order to enhance their intrinsic motivation to improve [2,10]. The module also acknowledges the challenges of receiving feedback given for learning purposes and a summative evaluation in the same session [17].

Core Section 3: Reflection and Self-assessment

This section prompts learners to answer open-ended questions and record their answers. Learners are asked to identify the strengths and the weaknesses they exhibited during their rotation, as per the commonly used Pendleton or “ask-tell-ask-tell” method [9]. This compels learners to reflect on their performance prior to entering the final feedback session. Learners are also asked to record what their learning goals were for the rotation and to elaborate a plan of action to improve one of their self-identified weaknesses. They are informed that goal-setting behavior in learners increases the effect of feedback [2]. Finally, learners are asked to reflect on their wellness state during the current rotation. This question was included because emotional reactions affect the recipient’s perception of the feedback and can lead to avoidance or discounting of feedback [18]. It also hints at the importance of metacognition capacities [11]. By identifying and acknowledging their emotions regarding the clinical rotation, learners might more easily recognize the impact those emotions can have on the feedback they are about to receive.

Initial user feedback

Based on the principles of Design-Based Research [19], the initial user feedback (i.e. learners) is considered a pre-research activity designed to improve an educational intervention before it is subjected to rigorous testing within an educational setting. During this phase, if necessary, the design team may make adjustments to the innovation as they are being used to account for these unanticipated conditions [20]. Consequently, this work was considered quality improvement work (pre-research) and our institution does not require a research ethics board review for quality improvement studies, as per Tri-Council Policy Statement, article 2.5 [21].

Therefore, we asked 18 third- and fourth-year medical students from the Université de Montréal to complete the learning application in an informal context, meaning they were not necessarily using the application before a planned feedback session. Participation was done on a voluntary basis. Following the completion of the module, learners were instructed to complete a short, anonymous feedback survey. The objective was to collect their comments regarding the application and highlight areas of improvement needed.

**Figure 2:**
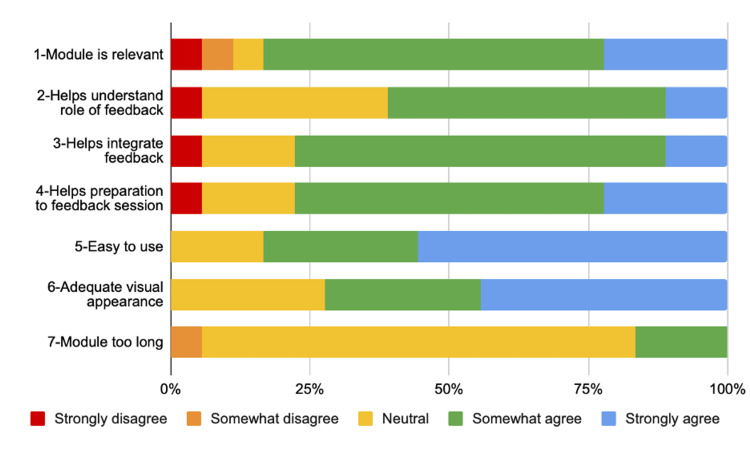
Results of Learner-Based Feedback Questionnaire

The feedback survey consisted of a questionnaire including items adapted from the Michigan Standard Simulation Experience Scale (MiSSES) questionnaire [22] and the System Usability Scale [23]. The items covered different aspects, including the application’s relevance, content, length, usability, and esthetics, and were rated using a 5-point Likert scale (Table 1). Two free-text questions were added to allow learners to provide general comments and suggestions for improvement. Likert-scale results are presented in Figure 2 and examples of free-text comments are available in Table 2.

**Table 1:** Learner-Based Feedback Questionnaire * Item adapted from the Michigan Standard Simulation Experience Scale (MiSSES) questionnaire; ** Item adapted from the System Usability Scale (SUS) questionnaire

1. The subject of the module is relevant.
Strongly disagree
Somewhat disagree
Neutral
Somewhat agree
Strongly agree
2. I believe the module will allow students to better understand the role of feedback in their medical careers.*
Strongly disagree
Somewhat disagree
Neutral
Somewhat agree
Strongly agree
3. I believe the module will allow students to be better equipped to properly integrate the feedback.*
Strongly disagree
Somewhat disagree
Neutral
Somewhat agree
Strongly agree
4. I believe the module will allow students to orient their personal reflection and arrive prepared for their feedback session.*
Strongly disagree
Somewhat disagree
Neutral
Somewhat agree
Strongly agree
5. The application is easy to use and navigate.**
Strongly disagree
Somewhat disagree
Neutral
Somewhat agree
Strongly agree
6. The application’s visual appearance is adequate.
Strongly disagree
Somewhat disagree
Neutral
Somewhat agree
Strongly agree
7. I believe the module’s length is ________.
Much too short
Too short
Adequate
Too long
Much too long
8. Suggestions for improvement*
9. General comments

**Table 2:** Examples of Free-Text Comments MS3: Third-year medical student; MS4: Fourth-year medical student; SMART: Specific, Measurable, Achievable, Realistic, Timely

Category	Comments
Content Improvement (elements to add)	“Preparing clerks for feedback they disagree with. How to advocate for themselves during a feedback session. How to provide constructive feedback on the rotation environment."
	“Rotation objectives should be available within the application (or through a link), as well as the rotation evaluation grid. Examples should be given of what is considered 'meeting expectations' for MS3 and MS4, for each evaluation criteria, to help students understand what is expected.”
	“It could be interesting to present a list of potential objectives, in which clerks could rank or select their top five objectives. Mention SMART objectives method.”
Format Improvement	“The introduction is a bit too long (two first sections), we lose focus.”
	“Possibly a bit long. For the section 'Understand the role of feedback', I believe medical students already understand quite well what that is. The 'Advice' section could be shortened in key points!”
	“Too many small sections in my opinion. I would rather have everything on one page, rather than clicking 'next'.”
Implementation Considerations	“This module could replace the mid-rotation self-assessment sheet (which needs to be completed in some rotations) but should not be added to it.”
	“I am wondering whether the best moment to complete this module is before the mid-rotation evaluation, or the final evaluation. Mid-rotation would be an interesting timing, since many questions refer to potential areas for improvement and strategies to improve. Completing the module before the final evaluation would therefore not allow enough time to actually apply those strategies.”
General Comments	“It is a very interesting application and the free-text questions are the most important part. I think this tool should be used in all rotations.”
	“Very relevant and a great reminder of the purpose of mid-rotation evaluation.”
	“Feedback sessions seem easy, but I often do not know what to say to the supervisor. The application is easy to use, simple, efficient, quick to complete, clear and precise. It allows us to reflect, in order to be better prepared to discuss with the supervisor, but also for ourselves.”
	“I believe that the problem is not necessarily that students do not know how to receive feedback. Speaking from experience, there are a lot of power issues, with some rotation supervisors being unable to provide detailed feedback or are not receptive to constructive comments provided by students in return. This makes it difficult to open up to them and have a discussion on our learning.”
	“The relevance is debatable. I think a 30 to 60 minutes interactive course at the beginning of the rotation would be more useful to reflect on ways to receive an evaluation, because it is indeed a source of stress for many.”

Relevance and Overall Appreciation

A vast majority of learners (83.3%) agreed or strongly agreed that the module was relevant. They generally felt that this module would help learners have a better understanding of the role of feedback in their medical career (61.1% of learners agreed/strongly agreed; 33.3% neutral). Moreover, the module was deemed useful to guide learners in their personal reflection by 77.8% of learners, and 77.8% believed it would help learners prepare for feedback sessions, facilitating better integration of comments received.

Free-text survey comments bear witness to learners’ appreciation of the module, with chosen qualifiers including “excellent”, “relevant”, “interesting”, and “efficient”. Two learners however shared diverging opinions. One believed an interactive course would be more appropriate than a mobile application. The second highlighted that the module's relevance was restricted since interactions during feedback sessions were rather limited by supervisors’ inability to provide detailed feedback, or their closedness to constructive comments on the learning environment or the rotation itself.

Content

The “Reflection and self-assessment” section was deemed the most important one, helping learners review their own performance and clearly identify their objectives. Different ideas emerged from the free-text comments in terms of additional content that could complement this section. These included listing potential objectives, to aid learners in pinpointing which ones they want to attain, or embedding the current rotation’s learning objectives, as well as examples of behaviors meeting the anticipated level of performance for each objective, to better define expectations. One learner also suggested adding advice on how to advocate for themselves if they disagree with the feedback received and how to provide constructive comments on the rotation environment.

Format

The application was considered easy to use and navigate (83.4%), and the visual appearance was judged adequate (72.2%). One learner however noted that the module could be divided into fewer sections. Most learners felt the module's length was appropriate (77.8%), although 16.7% felt it could be shortened. The first two sections (“Introduction” and “Understanding the role of feedback”) were identified as potentially too long because “medical students already understand (the role of feedback) quite well”, and one learner suggested summarizing the “Practical tips” section in key points.

Implementation Considerations

Although the application was designed to be completed before end-of-rotation summative evaluations, many learners highlighted that its benefit might be even greater when used before mid-rotation feedback. However, although not employed in every rotation, a mid-rotation self-evaluation sheet already exists in our institution. This sheet simply asks learners to identify their strengths and weaknesses, therefore some overlap with our application exists. This led some learners to specify that both of these tools should not be used at the same time.

## Discussion

Although there is growing literature on learner-related factors influencing feedback’s efficacy, there is currently no widely adopted curriculum or module available to directly address those factors. This technical report described the concept, design, and initial learner-based assessment of an innovative mobile application, available on computers, tablets, and phones, developed in order to bridge this gap. To our knowledge, this is the first application designed to improve medical learners’ reception of feedback.

Preliminary learners’ assessment revealed an overall positive response and supported the application’s relevance in a clinical setting. Minor improvements were suggested, including shortening the introduction, as well as the “Understanding the role of feedback” section. On the other hand, the “Reflection and self-assessment” section, deemed the most essential one, could be complemented by listing potential objectives to help learners identify which ones they want to prioritize. The objectives could be generic or correspond to objectives specific to the current rotation. The application will soon be modified accordingly.

The principal limitation of this application, which was highlighted by some learners, is the fact that it focuses only on the person receiving feedback, and therefore plays no role in helping supervisors provide constructive and effective feedback. Although learner-related factors are crucial, many supervisor-related factors preventing effective feedback in clinical settings were also identified in the literature. For example, end-of-rotation evaluation is perceived as less helpful than feedback sought by the learner notably because it often lacks specific comments [13]. Other identified barriers include time constraints and suboptimal feedback delivery by supervisors [12]. Such obstacles can potentially decrease the benefits arising from a learning opportunity, despite the learner’s best efforts. Thus, it is imperative to keep fostering a healthy learning environment and training supervisors on how to provide feedback. The online application focuses on learner-related factors that influence feedback efficacy and should be complementary to initiatives focusing on supervisor-related factors.

Future work will include the development of a second iteration of the application in response to the suggestions provided in our feedback survey. The application will then be tested in an authentic clinical setting, in order to analyze its true effects on the feedback process. The optimal timing to complete the application, whether before mid-rotation feedback or before the end-of-rotation summative evaluation, also needs to be clarified.

In addition to the pragmatic development of the application to help learners with receiving and integrating feedback, future work may also focus on more theoretical implications. Specifically, as described by Travares et al., feedback and debriefing may be merged under a common term referred to as learning conversations because of their conceptual and theoretical consistency [24]. Accepting this larger theoretical framework of learning conversations provides opportunities to explore similar approaches to prepare learners to engage in a more effective debriefing process.

## Conclusions

We developed an innovative application that aimed to help medical students benefit from feedback opportunities, by optimizing their reception of feedback. The learners’ initial positive response supports further efforts to refine and validate this application. Future steps include modifying the application based on learners’ comments, evaluating its efficacy in a real clinical setting, and clarifying whether it is most beneficial for mid-rotation or end-of-rotation feedback sessions.

## Appendices

Appendix 1 : Learning Module Content 

**Table 3:** Learning Module Content

Section	Content
Information	This module was developed by the medical education research group of Dr. Erica Patocskai, who is responsible for the Université de Montréal’s surgery clerkship rotation. The tool is destined for third and fourth-year medical students who complete clinical rotations and receive feedback from their clinician supervisors. The module takes a few minutes to complete and is designed to be completed on the same day as the final feedback session.
	The goals of this module are: 1. Help you better understand the role of feedback in your medical studies. 2. Better equip you to properly integrate the feedback. 3. Guide your personal reflection in order to prepare you for your feedback session.
Disclaimer	This module is 100% confidential and anonymous. The person responsible for your final evaluation may be aware of this module. However, neither your evaluator nor the Faculty of Medicine have access to your answer.
Understanding the Role of Feedback	First of all, what is feedback? Feedback is a complex process involving the learner and their mentors. Feedback allows learners to gain information on their current level of performance in a given skill and identify ways to improve. The learner integrates this information when they are practicing the skill and gradually improving. This process is repeated cyclically until the skill is mastered.
	For a third or fourth-year medical student, this means identifying their strengths and areas of improvement with the help of the residents and physicians supervising them, setting learning objectives and strategies to achieve them, then applying these in their upcoming clinical activities. The medical student will gladly note their gradual growth in the different clinical skills, knowing that this process will repeat itself all along their clerkship and residency.
	In summary, feedback allows students and residents to work towards their ultimate goal: becoming a competent physician!
Practical Tips to Optimize Feedback Session	1. You have the right to participate in the discussion. Ask questions about the feedback that is given to you. Make sure you have all the necessary information to understand your current level of performance and how you should be progressing. With your evaluator, search for concrete strategies to help you improve and achieve your goals.
	For example, if your evaluator tells you that you should improve your follow-up of hospitalized patients, you could ask them: “Could you clarify what is expected in terms of patient follow-up for a student of my level?” Furthermore, feel comfortable asking for more written comments to describe your performance in the rotation.
	2. Identify your primary goals. If you feel overwhelmed by your areas of improvement, identify one or two elements you want to improve first. You can focus on elements that are objective or modifiable. You will then be well-positioned to develop your strategy to improve these modifiable behaviours and you will note your progress in these areas.
	3. View the feedback in its context. The final feedback session is also an evaluation. Your evaluator is asked to evaluate each student fairly, amongst all students of the same level, across all training sites. Therefore, it is normal that most of your evaluation form states that you “meet expectations”. Focus on comparing your performance with yourself: identify your strengths and areas of improvement, and follow your progress from the mid-rotation evaluation to the end-of-rotation evaluation, and from one rotation to another.
Reflection and Self-assessment	This section is composed of five open-ended questions that will guide your personal reflection and will prepare you for your feedback session. It is probable that the physician you will meet during your feedback session will ask you similar questions. You can take this opportunity to verify if your self-assessment is realistic and adjust your perception accordingly.
	1. What were my goals for this rotation? Did I achieve them?
	2. What strengths did I demonstrate during this rotation?
	3. What weaknesses or areas of improvement did I identify during this rotation?
	Did you know? Learners who set goals linked to their feedback increase their chances of improving [1]. [1] Van de Ridder JM, McGaghie WC, Stokking KM, ten Cate OT. Variables that affect the process and outcome of feedback, relevant for medical training: a meta-review. Med Educ. 2015;49(7):658-73.
	4. Which area of improvement do I want to focus on? What strategy or action plan could help me achieve this goal?
	Did you know? Emotions play an important role in the feedback process and affect how we perceive the information we receive. Identifying these emotions can help you better understand your reaction to the feedback [2]. [2] Bing-You RG, Trowbridge RL. Why medical educators may be failing at feedback. Jama. 2009;302(12):1330-1.
	5. How would you rate your well-being during this rotation?
Conclusion	You have completed the feedback-preparedness module for medical students. You are now ready to actively participate in your feedback session and you are well-equipped to integrate the feedback in order to assist you in your continuous improvement.
